# The Centrality of Events, Religion, Spirituality, and Subjective Well-Being in Latin American Jewish Immigrants in Israel

**DOI:** 10.3389/fpsyg.2020.576402

**Published:** 2020-09-30

**Authors:** Hugo Simkin

**Affiliations:** ^1^ Carrera de Sociología, Facultad de Ciencias Sociales, Universidad de Buenos Aires, Buenos Aires, Argentina; ^2^ Department of Sociology and Anthropology, Faculty of Social Sciences, Tel Aviv University, Tel Aviv, Israel; ^3^ Consejo Nacional de Investigaciones Científicas y Técnicas (CONICET), Buenos Aires, Argentina

**Keywords:** migration, centrality of events, religion, spirituality, subjective well-being

## Abstract

This study aims to explore the impact of migration as a central event in personal identity, spirituality, and religiousness on subjective well-being (SWB). The sample was composed of 204 Latin American immigrants living in Israel, with ages ranging from 18 to 80 years (*M* = 48.76; *SD* = 15.36) across both sexes (Men = 34.8%; Women = 65.2%). The results show that, when analyzing the effects on SWB, Positive and Negative Affect, Centrality of Event, Religious Crisis, and Spiritual Transcendence present as the most relevant explanatory variables within the models. However, contrary to expectation, the present study identifies positive associations between the centrality of migration and SWB. Motivations for emigration should be explored in further studies as they could be mediating the relationship between centrality of events and SWB.

## Introduction

Since the creation of the State of Israel in 1948, about 92,000 Jews migrated from Latin America (Lesser, 2016) and well over half this group have come from Argentina (58%), with smaller groups from Brazil (11%), Uruguay (9%), Chile (6%), Mexico (4%), Colombia (3%), Venezuela (2%), and other countries (7%) (Babis, 2016). Although ideological, political, and religious reasons for *aliyah* have featured emphatically in the academic literature (Babis, 2016), Latin Americans’ motivations to migrate to Israel frequently include financial and political factors, and anti-Semitism (Rein, 2010, 2013; Siebzehner, 2010, 2016; Klor, 2016). For example, the cyclical behavior of the Argentine economy and the 2-year anti-Semitic campaign that followed the kidnapping of Eichmann contributed to a surge in the number of Argentine immigrants to Israel in 1963 (Rein, 2001) and, besides the impact of anti-Semitism on migrants’ decisions, most were seriously affected by the country’s economic fluctuations, recessions, and currency devaluations (Krupnik, 2020). Political persecution during military regimes in some Latin American countries, like Argentina and Chile, has also been flagged as factors that influenced this immigration during the 1970s (Babis, 2016). More recently, the 1992 attack on the Israeli Embassy in Buenos Aires, the 1994 bombing of the AMIA Jewish Community Center in Buenos Aires, and Argentina’s political and economic crisis between 1999 and 2002 have also been flagged among events that have led to Latin American emigration to Israel (Krupnik, 2011; Sznajder, 2015; Babis, 2016; Lesser, 2016).

### Migration, Trauma, and Well-Being

According to the literature, when migration is triggered by preservation motivation – when physical, social, and psychological security for self and family are motivators of emigration – it can be a stressful and traumatic event, and may have a negative impact on mental health (Finklestein and Solomon, 2009; Vathi and King, 2017). Migrants from socio-politically unstable countries may have been in situations where their lives or those of friends and family members, were in danger, so they not only have to negotiate the process of adaptation to a new social environment (Babis, 2016; Hashemi et al., 2019), but also may simultaneously experience vulnerability to various psychological symptoms related to higher levels of depression, post-traumatic stress disorder (PTSD), and lower levels of subjective and psychological well-being (Foster, 2001; Kalir, 2015; Tartakovsky et al., 2017). Gal (2020) has recently explored childhood experiences of anti-Semitism during Argentina’s last military dictatorship (1976–1983). She interviewed 15 participants, applying the narrative approach method and observations: the thematic textual analysis of these interviews centered on the behavioral and emotional expressions of these immigrants’ past experiences in their current lives as adult immigrants in Israel. The findings revealed that participants were exposed to severe manifestations of anti-Semitism during their childhood and that their present-day experiences include a variety of negative, long-term emotional and behavioral reactions.

### The Centrality of Traumatic Events in Personal Identity and Well-Being

Personal memories give meaning and structure to people’s life stories and help bolster individual identity (Baerger and McAdams, 1999; Watson and Dritschel, 2015). According to Berntsen and Rubin (2006), autobiographical memory is usually slanted toward positive life events, so that people tend to remember more positive than negative events from their lives, thus supporting a more positive view of themselves. A conventional life script contains more positive than negative events, often about such culturally expected role transitions as a graduation or a wedding (Berntsen and Rubin, 2006). However, for some individuals, a negative event perceived as traumatic, highly stressful, or shocking, like the loss of a loved one or a near-death experience, could, under certain circumstances, become pivotal. People who define specific stressful or traumatic events as being central to their identities believe that these events make their lives different from those of most other people, and that people who have not experienced these types of events think differently than they do (Berntsen and Rubin, 2006; Vermeulen et al., 2019). These memory characteristics are usually associated with feelings of being detached from other individuals within their own society, with implications for psychological well-being (Watson and Dritschel, 2015). Hence, if migrants’ traumatic memories form “turning points” in the organization of experiences, they may form a central component of personal identity and be harmful to mental health (Bernstein et al., 2003; Cerniglia and Cimino, 2012; Staugaard et al., 2015; Brooks et al., 2017). Results from the systematic review by Gehrt et al. (2018) of 92 publications show that centrality of event probes aspects of autobiographical memory that are of broad relevance to clinical disorders and have specific implications for PTSD.

### Religion, Spirituality, and Coping

In stressful times, some individuals tend to turn to religion for support, and although research shows that religion can be a positive force for mental health, it can also have a negative emotional impact in experiences of religious crisis, defined as conflicts related to religion, co-religionists, and relationships with God (Piedmont, 2012; Shilo et al., 2016; Abu-Raiya et al., 2020). Because of this, when triggered by Jewish identity, religion, and spirituality could be related to positive and negative coping mechanisms for post-migration stressors (Koenig et al., 2012; Rosmarin et al., 2017; Pirutinsky et al., 2019). Among other positive and negative issues, while religious affiliation and practice can provide social support for immigrants, they may also prompt feelings of rejection by the religious community when people are unable to live according to strict moral and religious standards (Piedmont, 2009; Dein et al., 2012; Weber and Pargament, 2014; Kim et al., 2020).

Regarding spirituality, while for some authors it may provide a strong sense of connectedness with others, which could help in coping with traumatic events (Braganza and Piedmont, 2015; Sigamoney, 2018; Khursheed and Shahnawaz, 2020), other authors argue that spiritual people without a religious framework are more vulnerable to developing mental disorders (King et al., 2013). Among other positive and negative issues, while spirituality could give people meaning and a sense of purpose (Piedmont, 2012; Le et al., 2019), harmful spirituality includes rigidity of belief, coercion, and the alienation of spirituality groups or cult members from outside supports (Galen, 2015; Kao et al., 2020).

### The Present Study

This study aims to explore relationships between centrality of event (migration) in personal identity, religion, spirituality, and subjective well-being (SWB). We postulated that, being central to identities, the experience of migration, spirituality, and religion, are associated with well-being.

## Materials and Methods

### Participants

The sample was composed of 204 Latin American immigrants living in Israel, with ages ranging from 18 to 80 years (*M* = 48.76; *SD* = 15.36), of both sexes (Men = 34.8%; Women = 65.2%). Participants were born in Argentina (54.3%), Bolivia (0.5%), Chile (7.1%), Colombia (17.3%), Costa Rica (1.5%), Cuba (0.5%), Ecuador (1.5%), Guatemala (0.5%), México (2%), Perú (4.6%), República Dominicana (0.5%), Uruguay (5.6%), and Venezuela (4.1%), all countries sharing a language and a broad social and cultural identity (Vergara et al., 2010; Campos-Winter, 2018). Migration dates ranged from 1963 to 2019 (*M* = 12.16 years; *SD* = 11.62 years).

### Measures

#### Brief Centrality of Event Scale

The Brief Centrality of Event Scale (CES; Berntsen and Rubin, 2006) is a seven-item self-administered questionnaire that gauges how central an event is to a person’s identity and life-story (e.g., “I feel that this event has become part of my identity”/“Siento que este evento se ha transformado en parte de mi identidad”). In this study, it was specified that participants should focus on the event of migration to Israel. Responses are given *via* a Likert-type scale with five anchors ranging from 1 = “totally disagree” to 5 = “totally agree.” For the purposes of the present study, a version was validated and adapted to the Argentine context by Simkin et al. (2017), which reported adequate internal consistency (*α* = 0.86) and fit statistics [CFI = 0.99, RMSEA = 0.6, and CI (0.03, 0.08)]. In the current study sample, CES has also shown adequate internal consistency (*ω* = 0.90) and fit statistics [CFI = 0.99, RMSEA = 0.05, and CI (0.0, 0.1)].

#### Affect Balance Scale

The Affect Balance Scale (ABS; Warr et al., 1983) is an 18-item self-administered questionnaire measuring both positive (e.g., “Have you felt very happy?”/“¿Te has sentido muy alegre?”) and negative (e.g., “Have you felt like crying?”/“¿Te has sentido con ganas de llorar?”) affective experiences. Responses are given *via* a Likert-type scale with five anchors ranging from 1 = “never” to 5 = “very frequently.” For the purposed of the present study, a version was validated and adapted to the Argentine context by Simkin et al. (2016), which reported adequate internal consistency for both positive (*α* = 0.77) and negative affect (*α* = 0.86), and fit statistics [CFI = 0.94, RMSEA = 0.5, and CI (0.04, 0.06)]. In the current sample, ABS has also shown adequate internal consistency for both positive (*ω* = 0.90) and negative affect (*ω* = 0.81), and fit statistics [CFI = 0.96, RMSEA = 0.07, and CI (0.06, 0.08)].

#### Satisfaction With Life Scale

Satisfaction With Life Scale (SWLS; Diener et al., 1985) is a five-item self-administered questionnaire that measures satisfaction with life (e.g., “The conditions of my life are excellent”/“Las condiciones de mi vida son excelentes”). Responses are given *via* a Likert-type scale with seven anchors ranging from 1 = “strongly disagree” to 7 = “strongly agree.” For the purposes of the present study, the version validated and adapted to the Argentine context by Moyano et al. (2013) was used, which reported adequate internal consistency (*α* = 0.75). In the current sample, SWLS also shown adequate internal consistency (*ω* = 0.90) and fit statistics [CFI = 0.99, RMSEA = 0.05, and CI (0.0, 0.1)].

#### Assessment of Spirituality and Religious Sentiments Scale

Assessment of Spirituality and Religious Sentiments Scale (ASPIRES-SF; Piedmont, 2010) is a 35-item self-report questionnaire that measures Religious Sentiments and Spiritual Transcendence. On the one hand, Religious Sentiment consists of two dimensions: Religious Involvement (e.g., “How often do you pray?”/“¿Cuán seguido reza?”) and Religious Crisis (e.g., “I feel that God is punishing me”/“Siento que Dios me está castigando”). Spiritual Transcendence, on the other, includes three further dimensions: Prayer Fulfillment (e.g., “I find inner strength and/or peace from my prayers or meditations”/“Encuentro fuerza interior y/o paz en mis rezos y/o meditaciones”), Universality (e.g., “I feel that on a higher level all of us share a common bond”/“Siento que en un nivel superior todos compartimos un vínculo común”), and connectedness (“Although they are dead, memories, and thoughts of some of my relatives continue to influence my current life”/“Aunque ya fallecidos, recuerdos y pensamientos de algunos de mis parientes continúan influenciando mi vida actual”). The version used in this study was adapted to the Argentine context by Simkin (2017), with reported adequate internal consistency for Religious Involvement (*α* = 0.84), Religious Crisis (*α* = 0.68), Prayer Fulfillment (*α* = 0.91), Universality (*α* = 0.76), and Connectedness (*α* = 0.57), and adequate fit statistics reported both for Religious Sentiments [CFI = 0.99, RMSEA = 0.037, and CI (0.000, 0.060)] and Spiritual Transcendence [CFI = 0.95, RMSEA = 0.069, and CI (0.062, 0.076)]. In the current study sample, ASPIRES also shown adequate internal consistency for Religious Involvement (*ω* = 0.95), Religious Crisis (*ω* = 0.91), Prayer Fulfillment (*ω* = 0.83), Universality (*ω* = 0.84), and Connectedness (*ω* = 0.64), and adequate fit statistics both for Religious Sentiments [CFI = 0.99, RMSEA = 0.007, CI (0.004, 0.009)] and Spiritual Transcendence [CFI = 0.97, RMSEA = 0.007, and CI (0.006, 0.008)].

### Procedure

A non-probability sampling was carried out to select the cases. Participants were invited to collaborate voluntarily, and their consent was obtained. It was explained to the respondents that the data gathered from the study would be used exclusively for scientific purposes according to the code of ethics established by the National Council for Scientific and Technical Research (CONICET; Res. D No. 2857/06), and under Argentina’s National Law 25,326.

### Data Analysis

Statistical analyses were performed using IBM SPSS Statistics 25 (Armonk, NY, United States) and EQS 6.4 (Byrne, 1994; Bentler, 2006) for Windows. Goodness of fit for the regression models considered *R* as an indicator of the effect size and *R* ^2^ adjusted as an indicator of total variance (Freiberg and Fernández Liporace, 2015; Darlington and Hayes, 2016; Schroeder et al., 2016). The models’ assumptions of multicollinearity, homoscedasticity of the residuals, and non-autocorrelation were tested to confirm its goodness of fit (Freiberg and Fernández Liporace, 2015). The Durbin-Watson statistic was used to examine the non-autocorrelation – with possible values ranging from 0 to 4, assuming the independence of the residuals, with values in this study lying between 1.30 and 1.75. Likewise, condition index and variance inflation factor (VIF) were used in the diagnosis of multicollinearity, the former being below 30 and the latter below 10.

## Results

A correlational analysis was conducted to examine the associations between the variables under study (Cohen et al., 1983; Curtis et al., 2016). The results shown in Table 1 suggest Centrality of Events is associated with Positive Affect, Satisfaction with Life, Religious Involvement, Religious Crisis, Prayer Fulfillment, Universality, and Spiritual Transcendence (Table 1).

**Table 1:** Correlations between variables of interest.

		1	2	3	4	5	6	7	8	9	10
1. CES	1									
2. NA	−0.120	1								
3. PA	0.533^**^	−0.373^**^	1							
4. SWL	−0.479^**^	−0.475^**^	0.699^**^	1						
5. RI	0.240^**^	0.008	0.186^**^	0.138^*^	1					
6. RC	−0.234^**^	0.284^**^	−0.211^**^	−0.253^**^	−0.312^**^	1				
7. C	0.115	0.062	0.134	0.076	0.143^*^	−0.019	1			
8. PF	0.264^**^	−0.027	0.227^**^	0.163^*^	0.847^**^	−0.353^**^	0.061	1		
9. U	0.295^**^	−0.133	0.339^**^	0.217^**^	0.562^**^	−0.379^**^	0.296^**^	0.657^**^	1	
10. ST	0.306^**^	−0.023	0.300^**^	0.204^**^	0.787^**^	−0.350^**^	0.321^**^	0.915^**^	0.804^**^	1

NA, Negative Affect; PA, Positive Affect; SWL, Satisfaction with Life; CES, Centrality Event Scale; ST, Spiritual Transcendence; RI, Religious Involvement; RC, Religious Crisis; PF, Prayer Fulfillment; C, Connectedness; U, Universality.^*^p < 0.05; ^**^p < 0.01.

Three regression models were subsequently tested, including Positive Affect, Negative Affect, and Satisfaction with Life as dependent variables, and Centrality of Event, Spiritual Transcendence, and Religious Crisis as independent variables.

A stepwise regression analysis with backward method was used to address the research questions. Three models were obtained, in which Negative Affect was predicted by Religious Crisis, Positive Affect was predicted by Centrality of Events and Spiritual Transcendence, and Satisfaction with Life was predicted by Centrality of Event and Religious Crisis (Berlanga-Silvente and Vilà-Baños, 2014; Hayes and Rockwood, 2017) (Table 2).

**Table 2:** Goodness of fit for regression models.

	*R*	*R* ^2^	*R* ^2^ adjusted	SE	Durbin-Watson
NA	0.284	0.081	0.076	6.96	1.805
PA	0.552	0.305	0.298	6.01	1.839
SWL	0.500	0.250	0.243	4.07	2.076

NA, Negative Affect; PA, Positive Affect; SWL, Satisfaction with Life.

As can be seen in Tables 2 and 3, the model’s goodness of fit has been verified (Table 3).

**Table 3:** Condition index – multicollinearity.

		*Condition index*	*VIF*
NA	RC	5.102	1.000
PA	CES	8.916	1.103
	ST	13.295	1.103
SWL	CES	4.949	1.058
	RC	14.672	1.058

NA, Negative Affect; PA, Positive Affect; SWL, Satisfaction with Life; CES, Centrality Event Scale; RC, Religious Crisis; ST, Spiritual Transcendence.

The results show that, when analyzing the effects on SWB, Positive Affect, and Negative Affect, and Centrality of Event, Religious Crisis, and Spiritual Transcendence appear as the most relevant explanatory variables in the models (Table 4).

**Table 4:** Regression analysis for Negative Affect, Positive Affect, and Satisfaction with Life.

		*B*	*IC* 95%	*SE*	*Beta*	*t*	*Sig.*
NA	(constant)	20.334	[17.784; 22.885]	1.294		15.720	0.000
	RC	0.722	[0.384; 1.061]	0.172	0.284	4.210	0.000
PA	(constant)	10.622	[5.650; 15.593]	2.521		4.213	0.000
	CES	0.635	[0.476; 0.794]	0.080	0.487	7.890	0.000
	ST	0.067	[0.013; 0.121]	0.027	0.151	2.439	0.016
SWL	(constant)	8.065	[4.300; 11.829]	1.909		4.224	0.000
	CES	0.378	[0.273; 0.484]	0.053	0.444	7.070	0.000
	RC	−0.245	[−0.448; −0.041]	0.103	−0.149	−2.369	0.019

NA, Negative Affect; PA, Positive Affect; SWL, Satisfaction with Life; CES, Centrality Event Scale; ST, Spiritual Transcendence; RC, Religious Crisis.

Finally, the relations between Centrality of Events, Religion, Spirituality, and SWB in Latin American Jewish Immigrants in Israel were tested using structural equation modeling, evaluated from the CFA model fitting solution by the Standardized Root Mean Squared Residual (SRMR), as it is suggested for continuum variables (Shi et al., 2018; Pavlov et al., 2020). The results indicated a good model fit (Table 5).

**Table 5:** Goodness-of-fit.

	*X* ^2^_(*gl*)_	*CFI*	*IFI*	*NFI*	*GFI*	*SRMR*
Model	19.197_(4)_	0.965	0.95	0.94	0.97	0.046

CFI, comparative fit index; IFI, incremental fit index; NFI, (Non) normed fit index; GFI, goodness of fit index; SRMR, standardized root mean squared residual.

**Figure 1:**
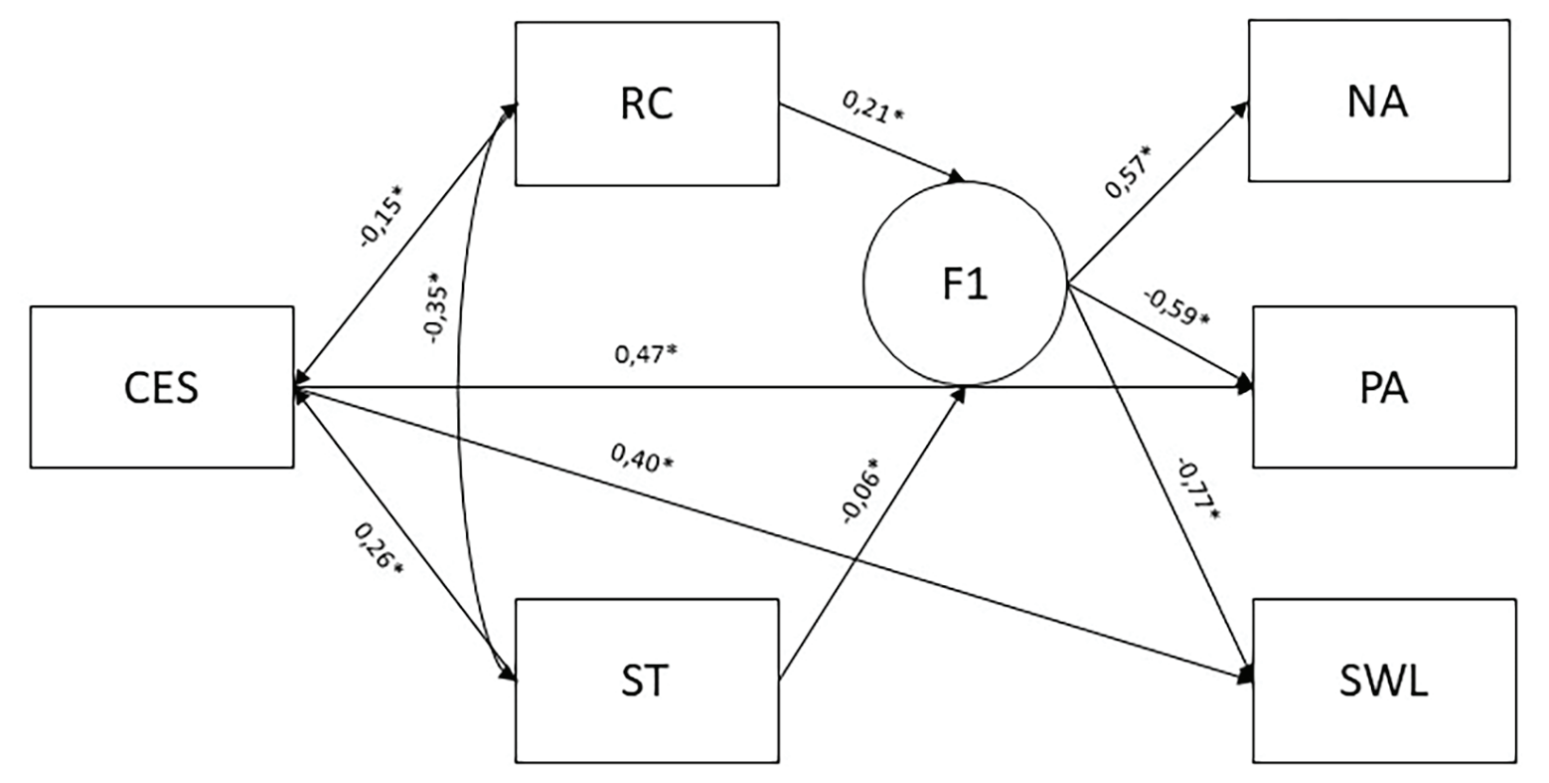
Structural model of Spiritual Transcendence, Religious Crisis, and Centrality of Event predicting Subjective Well-Being, with standardized estimates. NA, Negative Affect; PA, Positive Affect; SWL, Satisfaction with Life; CES, Centrality Event Scale; ST, Spiritual Transcendence; RC, Religious Crisis.

Figure 1 represents the path model and the different loadings among each latent construct.

Concordant with the regression analyses, Centrality of Event, Religious Crisis, and Spiritual Transcendence appears as the most relevant variables explaining SWB – both Positive and Negative Affects.

## Discussion

This study aimed to explore the impact of migration as a central event in Personal Identity, Spirituality, and Religiousness on SWB. First, our research contributes both to the literature on psychological studies of migration and the psychology of religion.

For psychological research on migration, Centrality of Event plays a significant role in a deeper understanding of the relevance of migration experiences in immigrants’ personal identities. This could partially explain the associations between migration and well-being. Indeed, more research on the relationship between migration and SWB is needed, given that most studies in the field focus on anxiety, depression, or PTSD, as the deficit model that has dominated psychology for decades (Cobb et al., 2019). It is therefore necessary to consider the positive effects of migration on individuals, which can contribute to the development of different facets of identity and psychological well-being. Migration can contribute to personal growth and the achievement of new life opportunities. If the early stages can lead to high levels of anxiety about uncertainty at the same time, they can promote new life projects (Bobowik et al., 2015).

For the psychology of religion – and especially for lines of research focusing on the relationship between religion and SWB (Demir, 2019) – migration to Israel is also a relevant topic, both because of the role of spiritual and religious beliefs linked to the process of migration to Israel, and the stressful conditions in migration processes that could be harmful to well-being.

### Centrality of Event and Subjective Well-Being

The results show migration to Israel to be a central event in the identities of Jewish Latin American immigrants. However, contrary to expectations, the present study has identified positive associations between the centrality of migration and SWB. According to the literature on the latter, traumatic and strongly negative events are not as frequently experienced as positively or neutrally perceived events, except for those who experience extremely negative life circumstances (Diener and Diener, 1996; Diener et al., 2018). In this light, “positive” and “negative” perceptions of migration to Israel as a central event may be mediated by motivations for emigration. As mentioned above, ideological and religious reasons have been mentioned among Latin Americans’ motivations for emigration to Israel alongside anti-Semitism and financial and political factors (Rein, 2010, 2013; Siebzehner, 2010, 2016; Klor, 2016). Similar motivations were identified by Tartakovsky and Schwartz (2001) as they developed the Motivation for Emigration Scale, focused on Russian Migrants to Israel. Hence, as there are no precedents for similar measures among Latin American migrants, new scales are needed to advance along this line of research. Furthermore, for a deeper understanding of motivations behind emigration, it is also important to explore not only motivations behind emigrations but also the historical events that have triggered them, specifically for those who emigrate because of anti-Semitism or for political or economic reasons.

An understanding of Latin Americans’ motivations for emigration could go some way to explaining the relationship between the centrality of migration and SWB, but there are other factors not yet properly studied within social psychology that are also relevant to this line of research. As Babis (2016) has pointed out, post-migration stressors for Latin Americans are strong and go beyond language barriers: the *integration/isolation* process among immigrants is relevant to the community as a whole. Even though acculturation has been widely studied among immigrants in Israel, most of the research has focused on Russians (Roccas et al., 2000; Tartakovsky, 2012) and Africans (Nakash et al., 2015, 2016). Acculturation is particularly relevant in Israel, as intergroup conflict is high in that country (Ditlmann and Samii, 2016; Dugas et al., 2018). Exploring Latin Americans’ perceptions of intergroup conflict in Israel could also be relevant to understanding the relationships between centrality of events, acculturation, and SWB.

Finally, as Argentines are the largest groups among Latin Americans, most research focuses on them. Future studies should focus on identifying the singularities of migratory experiences according to the specificities of each country.

### Religion, Spirituality, and Subjective Well-Being

As expected, where religion and spirituality are concerned, Religious Crisis and Spiritual Transcendence played an important role in explaining Satisfaction with Life, Positive Affect, and Negative Affect (Piedmont, 2012; Piedmont and Friedman, 2012). Because of the study’s small sample size (Kline, 2016; Goodboy and Kline, 2017), future studies should explore how motivations for emigration, centrality of events, spiritualty, religion, and well-being interact with each other within larger samples (N > 400; Kline, 2016; Medrano and Muñoz-Navarro, 2017).

## Conclusion

Migration to Israel could be considered a Central Event in Migrants’ Identities, having either a positive or negative impact on well-being. Future research is needed to elucidate what variables might be mediating this association. First, this study has only focused on SWB, but “negative” mental health variables such as depression, anxiety, and PTSD should be explored as much as “positive” mental health variables such as Psychological and SWB (Cobb et al., 2019). Second, motivations for emigration should be explored as they may be mediating the relationship between centrality of events and mental health. Third, other variables should be considered within this model. Acculturation and perception of intergroup conflict may be two of the most important related variables. Fourth, religion and spirituality play a specific role in the specific case of migration to Israel and await further assessment. Last, as most studies among Latin Americans focus on Argentines, more research is needed to explore other countries’ migration singularities.

## Data Availability Statement

The raw data supporting the conclusions of this article will be made available by the authors, without undue reservation.

## Ethics Statement

The studies involving human participants were reviewed and approved by Universidad de Buenos Aires, ethics committee. The patients/participants provided their written informed consent to participate in this study.

## Author Contributions

HS devised the structure, analyzed the literature, ran the data analysis, and wrote the manuscript.

### Conflict of Interest

The author declares that the research was conducted in the absence of any commercial or financial relationships that could be construed as a potential conflict of interest.
